# Peptide-Based Immunotherapeutics and Vaccines

**DOI:** 10.1155/2014/256784

**Published:** 2014-02-04

**Authors:** Pedro A. Reche, Enrique Fernandez-Caldas, Darren R. Flower, Masha Fridkis-Hareli, Yoshihiko Hoshino

**Affiliations:** ^1^Departamento de Microbiología I, Inmunología, Facultad de Medicina, Universidad Complutense, 28040 Madrid, Spain; ^2^Inmunotek SL, Alcalá de Henares, 28805 Madrid, Spain; ^3^School of Life and Health Sciences, Aston University, Birmingham B4 7ET, UK; ^4^ATR, LLC, Worcester, MA 01606, USA; ^5^Department of Mycobacteriology, National Institute of Infectious Diseases, Higashi-Murayama, Tokyo 189-0002, Japan

The discovery of small molecule and biologic drugs offers treatments applicable to a wide variety of infectious and chronic human diseases. Antibiotics have, since World War II, been the weapons of choice in humanity's on-going battle with pathogenic bacteria, and more recently the discovery of antiviral drugs has been an area of active endeavor. Yet the emergence of multiresistant bacteria, as well as the continuing prevalence of antigenic drift in viruses, has shown the fundamental limitations of postinfection drug therapy. Fortunately, the scientific exploitation of host immunity provides an even more powerful approach to forestalling infection and counteracting chronic disease states. The supreme embodiment of this is the vaccine. Indeed, prophylactic, and to a lesser extent therapeutic, vaccines continue to be the most cost-effective and efficient alternative to other medicines for the treatment and prevention of infectious diseases, cancer, and allergy conditions. Importantly, unlike drug-based medicines, vaccines are primarily curative not palliative remedies.

Vaccines work by inducing profound changes in the cellular components of adaptive immunity, comprising T- and B-cells. The first vaccines targeted infections pathogens and were composed of killed or attenuated pathogens. However, our emerging understanding of antigen presentation and subsequent recognition has enabled the development of several new types of vaccine, including those based on single proteins or on synthetic peptides encompassing many B- and T-cell epitopes [1]. Peptide-based epitope ensemble vaccines offer several potential advantages over traditional whole-organism vaccines. Chiefly, they allow the immune response to focus solely on relevant epitopes, avoiding those that lead to nonprotective responses, immune evasion, or unwanted side effects, such as autoimmunity. Consequently, peptide-based vaccines in particular offer renewed hope for the treatment and prevention of chronic infectious diseases such as those caused by hepatitis C virus and cancer [2, 3]. Likewise, peptide-based vaccines can also be used as safer ways of inducing allergen specific tolerance [3].

The design of peptide-based vaccines takes advantage of an emergent computational paradigm that couples immunoinformatic prediction with rigorous experimental validation, thus facilitating the identification of epitopes within protein antigens [1]. Amongst extant prediction technologies, the most fruitful has been data-driven prediction of T-cell epitopes. T-cell epitope prediction relies primarily on the anticipation of peptide-binding to MHC molecules [4] and many methods drawn from bioinformatics and/or chemoinformatics are known to produce satisfactory results [5, 6].

However, development of effective peptide-based vaccines requires science to overcome a number of significant difficulties, including identifying optimal delivery routes, overcoming the low intrinsic immunogenicity of individual peptides, and the task of combining different types of epitopes to engage the humoral and cellular arms of the adaptive immune response properly, as well as compensating for the poor population coverage of individual T-cell epitopes due to MHC restriction. On a more technical level, certain antigenic epitopes, specifically B-cell epitopes or antibody determinants, are conformational and need to be mimicked by linear sequences or placed onto suitable spatial frameworks. Moreover, methods for predicting B-cell epitopes need to be improved as they are notoriously unreliable in predicting both linear [7] and conformational [8, 9] B-cell epitopes.

In this special issue on peptide-based vaccines, we have gathered together a varied array of cutting-edge reports and reviews detailing several important and innovative approaches to the design and discovery of epitope-based vaccines. Articles range from reports on new computational methods for epitope prediction and selection for vaccine design to clinical trials using peptide-based vaccines.

We hope that readers will find this special issue not only interesting but also enlightening, exciting, and inspiring. As more and more pathogen genomes are revealed and our understanding of fundamental immunological mechanisms deepens, it becomes ever clearer that the only tractable approach to vaccine discovery and design is through the interplay of *in silico* with *in vitro* and *in vivo* techniques. Indeed, the combination of computational and experimental epitope mapping offers many tools and techniques appropriate to the identification and optimization of vaccine candidates. As this special issue demonstrates so well, such methodology is fast becoming one of the most pivotal tools currently available to vaccine developers; and it foreshadows the day when this variety of approaches will fully synergize to speed up the development of powerfully efficacious vaccines against both infective and chronic disease states.

*Pedro A. Reche* *Pedro A. Reche* *Enrique Fernandez-Caldas* *Enrique Fernandez-Caldas* *Darren R. Flower* *Darren R. Flower* *Masha Fridkis-Hareli* *Masha Fridkis-Hareli* *Yoshihiko Hoshino* *Yoshihiko Hoshino*
